# Bilateral Brachial Artery Disease Presenting with Features of Raynaud's Phenomenon: A Case Report and Review of the Literature

**DOI:** 10.1155/2017/7461082

**Published:** 2017-07-10

**Authors:** Karan Seegobin, Brittany Lyons, Satish Maharaj, Cherisse Baldeo, Pramod Reddy, James Cunningham

**Affiliations:** ^1^Department of Internal Medicine, University of Florida College of Medicine, Jacksonville, FL 32209, USA; ^2^Department of Interventional Radiology, University of Florida College of Medicine, Jacksonville, FL 32209, USA

## Abstract

**Objective:**

To present a case of bilateral brachial artery disease presenting with features of Raynaud's phenomenon which was successfully treated with angioplasty and stenting, together with a review of the relevant literature.

**Case:**

A 71-year-old female presented with a one-year history of intermittent pallor of both hands precipitated with cold objects. On examination, bilateral radial pulses were reduced. Prior photos showed pallor of the distal aspect of both palms. Angiogram showed high grade stenosis of the right brachial artery and focal occlusion with likely dissection of the left brachial artery. She underwent angioplasty and stenting for both lesions. She was asymptomatic without further episodes of Raynaud's phenomenon after five months on dual antiplatelet therapy. Upper-extremity vascular stenosis is uncommon. Structural changes in the vessel wall can cause vasospastic attacks, a mechanism described in secondary Raynaud's phenomenon. We hypothesize that these attacks may have been precipitated by the bilateral brachial artery disease. Furthermore, resolution of the symptoms after stent further supports our theory.

**Conclusion:**

Bilateral brachial artery disease is uncommon. Physicians should consider this in patients presenting with Raynaud's phenomenon. Brachial artery stenosis and occlusion is a treatable disease with good symptomatic outcomes after angioplasty and stenting.

## 1. Introduction

Brachial artery disease is uncommon [1]. Because it is rare, the optimal treatment strategy for brachial artery stenosis remains uncertain [1]. Endovascular treatment, with angioplasty or stenting, has been successfully performed for subclavian and below the elbow diseases; however, there is a lack of data regarding the treatment of brachial artery disease [2]. Here we describe a case of bilateral brachial artery disease presenting with features of Raynaud's phenomenon which was successfully treated with angioplasty and stenting. We further review and discuss the relevant literature.

## 2. Case

A 71-year-old female with a history of peripheral neuropathy of unknown etiology presented with a one-year history of intermittent pallor of both hands which was precipitated with cold objects. She fell from a chair onto her elbows and hands one year earlier, but no fractures or dislocations were sustained. There was no history of rest or claudication pain and no prior instrumentation of the vessels. She had no family history of autoimmune or hypercoagulable conditions and no history of smoking, alcohol, or illicit drug use.

**Figure 1:**
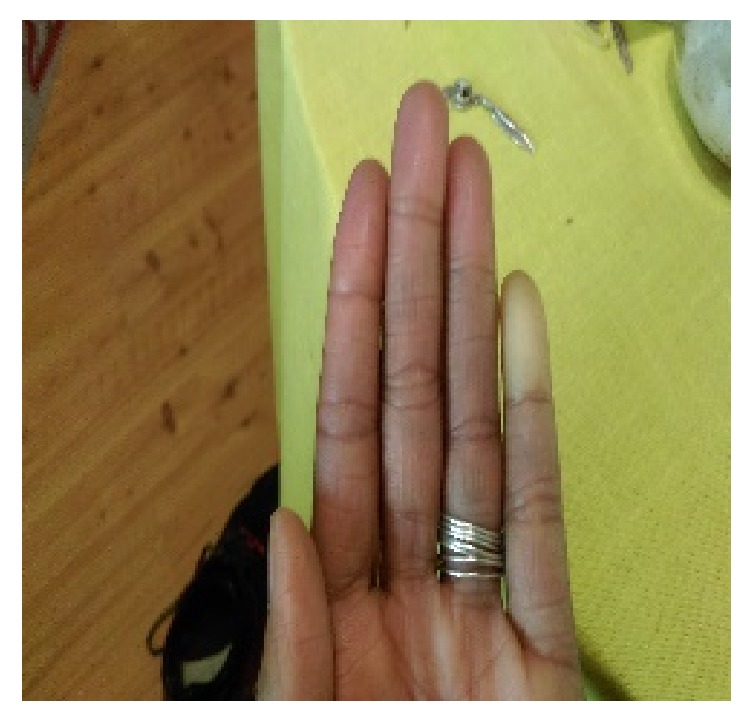
Pallor of the distal fingers of the left hand.

**Figure 2:**
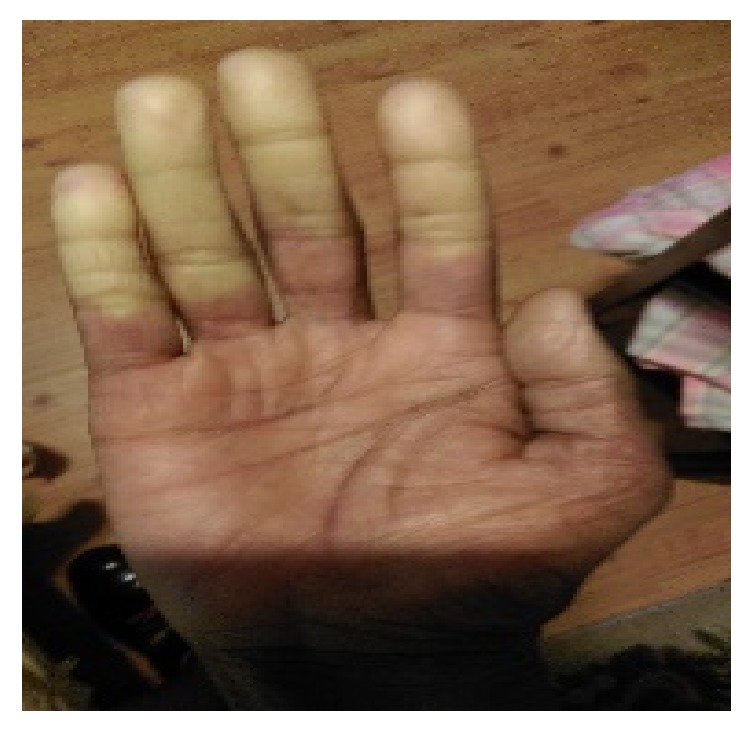
Pallor of the distal fingers of the right hand.

**Figure 3:**
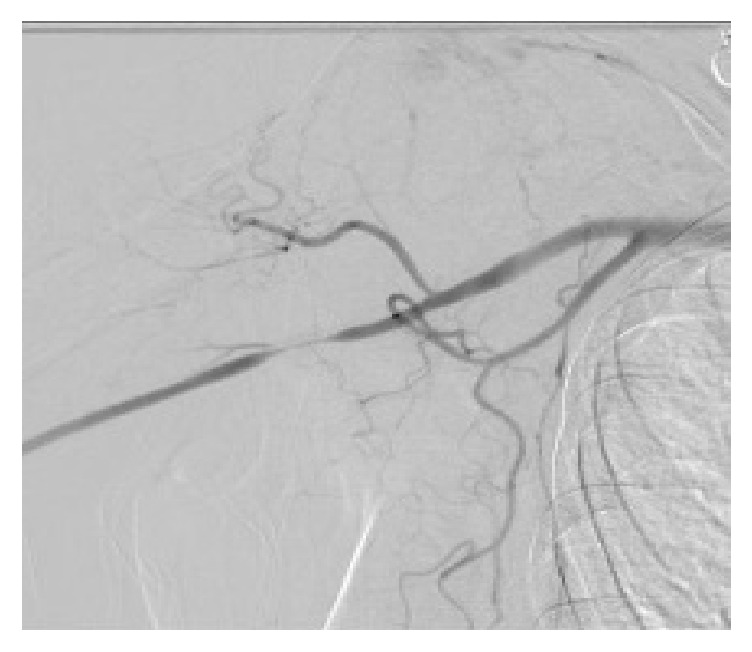
Tapered high grade stenosis of the proximal right brachial artery with collateral muscular branches.

**Figure 4:**
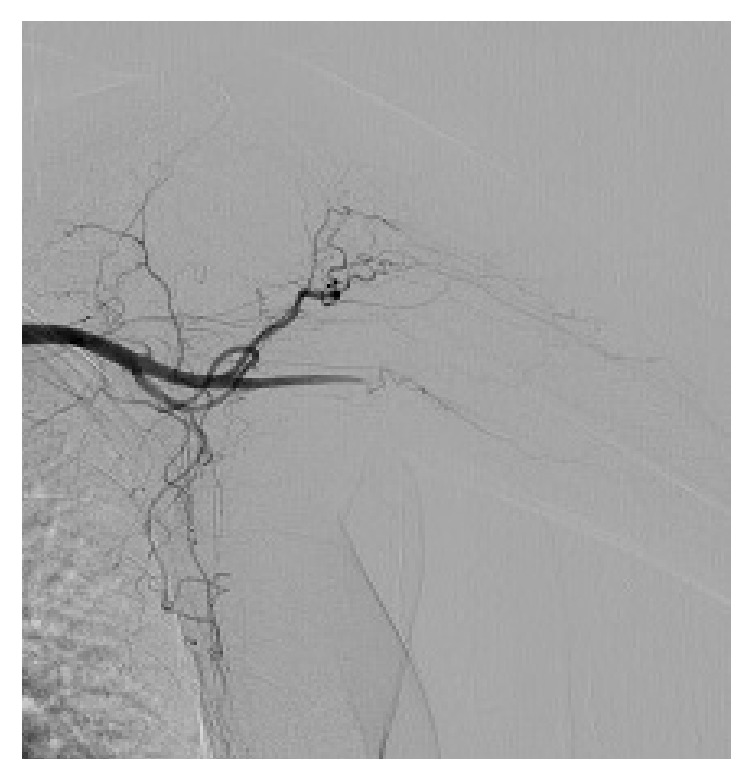
Left brachial artery with a short focal tapered occlusion, with likely dissection and collateral muscular branches.

On examination, her blood pressure was 120/60 mmHg on the right arm and 100/50 mmHg on the left, pulse 86 beats per minute, and respiratory rate 18 breaths per minute. Her radial pulses were reduced bilaterally. There were no sensory deficits in her bilateral upper extremities and no palmar atrophy. Other aspects of her clinical examination were unremarkable. There was no pallor or cyanosis of the palms of her hand at the encounter. However, prior photos taken showed marked pallor of the distal fingers of the palms of both hands (Figures 1 and 2). Laboratory investigations including autoimmune screen, ESR, CRP, and prothrombotic panels were unremarkable. HIV testing was negative. Echocardiography was normal with an ejection fraction of 65–70%. CT angiography showed a focal right brachial artery stenosis and a short segment 2 cm occlusion of left brachial artery with distal reconstitution by muscular collateral branches. She further underwent conventional angiography which showed tapered high grade stenosis of the proximal right brachial artery (Figure 3) and on the left a short focal tapered occlusion, with likely dissection of the left brachial artery reconstituted with collateral muscular branches (Figure 4).

**Figure 5:**
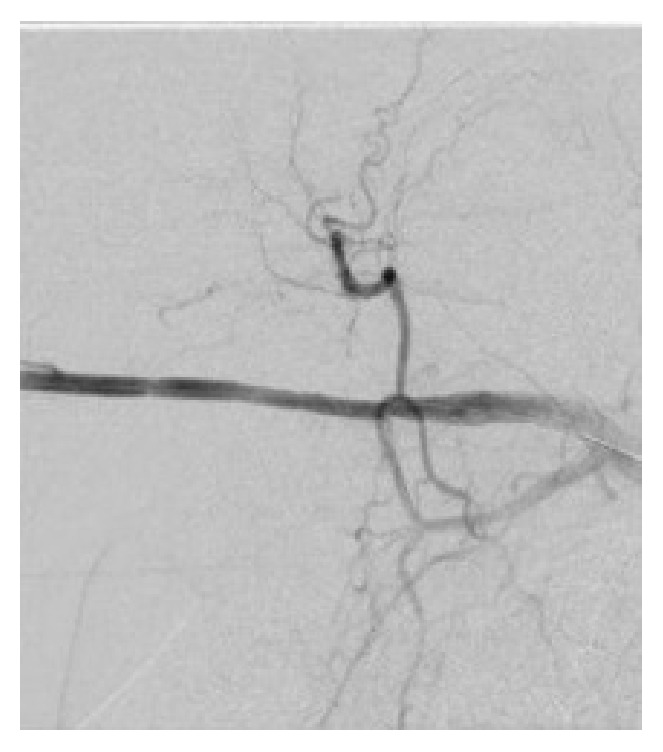
Repeat angiogram of the right brachial artery showed no residual stenosis after balloon angioplasty and stenting.

**Figure 6:**
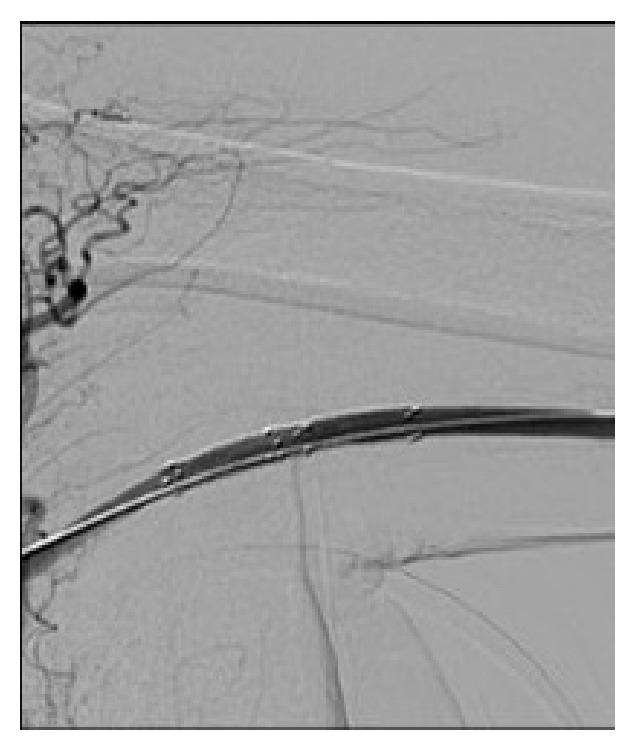
Repeat angiogram of the left brachial artery showed no residual stenosis after balloon angioplasty and stenting.

Treatment was undertaken on the right with low pressure balloon angioplasty across the affected segment. After angioplasty there was modest improvement in luminal diameter with no extravasation of contrast and a 5 mm × 5 cm Viabahn covered stent was inserted and then postdilated with 4 mm mustang balloon. Repeat angiography showed excellent positioning of the stent and no residual stenosis. Runoff angiogram showed widely patent runoff arteries (Figure 5). A stent was applied to the focal dissection on the left and a 5 mm mustang angioplasty balloon was advanced across the occluded segment and used for low pressure balloon angioplasty. The occluded segment was stented with 2 overlapping stents, 5 mm × 2.5 cm Viabahn stents. The stents were postdilated with a 5 mm mustang balloon. Poststent angiogram showed good poststenting positioning and no residual stenosis and unimpeded flow of contrast to the hand (Figure 6). There was no evidence of embolic phenomenon. There were no postprocedural complications and dual antiplatelet therapy with aspirin and clopidogrel was commenced. Five months later the patient remained asymptomatic without further episodes of hand pallor.

## 3. Discussion

Chronic upper limb ischemia is uncommon and is most often secondary to subclavian artery stenosis [1]. Because of significant collateral circulation, limb loss is rare and surgical intervention is infrequent, accounting for 4% of all vascular surgical procedures [2]. Furthermore, brachial artery stenosis is a rare phenomenon often associated with atherosclerotic disease, giant cell arteritis, fibromuscular dysplasia, trauma, and crutch related injuries [3, 4]. Brachial artery stenosis accounts for approximately 12% of symptomatic upper-extremity ischemia [1]. Upper-extremity ischemic symptoms are uncommon and typically occur only during upper-extremity exercise [5]; our patient on the other hand experienced symptoms with exposure to cold objects and did not experience pain with exercise. This could have been due to the presence of collateral vessels to perfuse her upper extremities. The rare occurrence of symptomatic distal upper-extremity ischemia compared with that of the lower extremity may be explained by rich collateral networks and small muscle mass [6]. Nevertheless, when occlusive disease and lack of collateral circulation are severe enough to cause symptoms and signs of ischemia, the degree of functional loss and pain experienced by the patient can be as devastating as that resulting from lower-extremity ischemia [6, 7]. Reports in this patient population indicate that symptomatic ischemia developed at the following rates: 10% to 25% in brachiocephalic and basilic level; 4% to 6% at forearm level; and 1% to 2% at radiocephalic level of forearm [6]. Currently, there is no guideline available for the treatment of upper-extremity ischemic events, except recommendations for specific cases [6].

The management of lower-extremity arterial ischemia has been extensively studied [6]. However, the management method of upper-extremity ischemia has not received as much attention [6]. A postulated reason for this relative lack of literature may be that upper-extremity ischemia is a rare event, regardless of the etiology [6].

Intervention is generally reserved for the treatment of symptomatic patients [5]. Before the spread of endovascular techniques for the treatment of vascular stenosis, symptomatic upper-extremity ischemia was managed with surgical revascularization [2]. Subclavian stenosis was usually managed with axilloaxillary bypass, carotid subclavian bypass, or subclavian artery transposition, with good results [2]. Although there are not many published studies of upper limb revascularization, brachial artery and forearm disease were also managed with surgical bypass [2]. Surgical excision of the abnormal right brachial artery with reconstruction and angioplasty has had success in patients with brachial artery disease related to fibromuscular dysplasia [8, 9]. Roddy et al. described the results of 61 bypass grafts in patients with atherosclerotic disease of the brachial artery [10]. Most patients were female and smokers and presented with rest pain and only 13% with tissue lost [10]. Good outcomes were observed with 1-year patency of 90.5% and limb salvage of 100% [10]. Furthermore, these open surgical techniques have been studied with reports of patency ranging from 48% to 100% at different follow-up intervals [6].

Percutaneous angioplasty (PTA) is another option in upper-extremity vascular disease [6]. After its introduction in the 1960s, there have been marked improvements and sophistication of devices commonly used today [6]. It brings many advantages which include less time and can be done with mild sedation and local anaesthesia; it costs substantially less and can be repeated with minimal difficulty if needed [6]. It also allows surgeons to gain access to regions that are impossible to properly explore with open procedures [6]. Postoperatively, patients often experience less pain compared with an open procedure, and recovery is shorter as well [6]. In the chronic renal disease population with stenosis of hemodialysis access grafts in the forearm, there have been reports of successful restoration of blood flow with balloon angioplasty [6]. Furthermore, several cases of nonatherosclerotic upper-extremity vascular disease of the brachial arteries were successfully treated with balloon angioplasty [6].

Now, subclavian stenting is being performed for atherosclerotic stenosis with good results, with reported technical success and primary patency rates of 98.3% and 84%, respectively [2]. In cases of subclavian artery stenosis balloon angioplasty is generally required prior to stenting [5].

Subclavian artery occlusions represent a more challenging technical subset [5]. The evolution in technique from angioplasty alone to angioplasty with provisional stenting and finally to angioplasty and stenting in all cases reflects the improved outcomes achieved with stenting [5]. The technical success rate in contemporary series approaches 100% [5]. The success rate for total occlusions remains significantly less but has dramatically improved over the last decade and can approach 90% for experienced operators [5].

The optimal treatment strategy for brachial artery stenosis remains uncertain. There is a lack of report regarding the treatment [1]. Efficacy of angioplasty in upper-extremity occlusive disease has been studied mostly in the innominate, subclavian, and axillary arteries [6]. Although most authors agree that success rate largely depends on severity, pathology, and location of the lesion, in general PTA gives comparable, if not better, results compared with conventional open surgery [6].

Endovascular treatment, with angioplasty or stenting, has been successfully performed for subclavian and below the elbow diseases; however [2] percutaneous transluminal angioplasty and vascular stent treatment has had success in axillobrachial artery stenosis from crutch related injuries and in patients with brachial artery occlusion [2, 4]. Furthermore intra-arterial infusion of thrombolytic and percutaneous transluminal balloon angioplasty has had success in treatment of occlusion with stenosis of brachial artery related to fibromuscular dysplasia [11].

Our case was unique in that there was dissection of the left brachial artery complicated with occlusion and high grade stenosis on the right brachial artery. Both angioplasty and stenting were employed in our case with good outcomes 5 months later.

Access-site complications are the most common serious adverse events reported (0%–7%) and are closely related to the use of the brachial artery for access [5]. Complications include hematoma formation, thrombosis, and pseudoaneurysm formation [5]. Stent migration is a real but uncommon complication [5]. The major long-term risk of subclavian artery intervention is restenosis [5]. After five months our patient remained free of these complications. Stenting appears to have significantly reduced the rate of restenosis, from ~15%–20% with angioplasty to 0%–10% [5]. We have not found any underlying cause for the bilateral brachial pathologies in our case; however one possibility is that it could have been precipitated by her fall in the past. Dissection can occur following minimal trauma; however it is a rare entity and has been reported in cases with subclavian dissection [12]. Several authors reported that elbow fractures are associated with brachial artery injury [13]. Furthermore brachial artery stenosis has been reported with a history of elbow fracture [13]. Our patient fell onto her hands and elbows; however she did not sustain any fractures from her fall. We postulate this as a possible cause of her bilateral brachial artery disease.

Structural changes in the vessel wall due to organized thrombus or extravascular compression can cause vasospastic attacks, a mechanism described in secondary Raynaud's phenomenon [14]. It is a vasospasm that occurs primarily as a result of sensitivity to cold temperatures or emotional stress [15]. Arteries of the upper extremity are more responsive to sympathetic tone compared with those of the lower extremity [6]. Raynaud's phenomenon is associated with distal digital colour changes that progress from white to blue to red, representing the initial ischemia from the vasospasm (white), the subsequent slow circulation leading to increased amount of deoxygenated blood (blue), and the final hyperemic state after vessel dilation (red). All three stages of colour change do not need to be present in order to diagnose Raynaud's phenomenon [16]. Our patient experienced this phenomenon from exposure to cold objects. We hypothesize that vasospastic attacks may have been precipitated by the bilateral brachial artery disease. Furthermore, resolution of the symptoms after stent further supports our theory. Arterial thrombosis has been reported as a complication of dissection [17]; we suspect the dissection in this case could be the underlying cause of thrombus formation.

## 4. Conclusion

Bilateral brachial artery disease is uncommon and can occur following minimal trauma. Physicians should consider this in patients presenting with Raynaud's phenomenon. Brachial artery stenosis and occlusion is a treatable disease with good symptomatic outcomes from angioplasty and stenting.
